# Impact of infratentorial location on survival after surgical resection of brain metastases: a multicenter retrospective study

**DOI:** 10.1093/noajnl/vdag060

**Published:** 2026-02-28

**Authors:** Artem Rafaelian, Matthias Schneider, Daniel Dubinski, David Wasilewski, Emanuela S Abdulrahman-Truta, Anna-Laura Potthoff, Hartmut Vatter, Silvia Duran-Hernandez, Julia Onken, Peter Vajkoczy, Thomas M Freiman, Florian Gessler, Sae-Yeon Won

**Affiliations:** Department of Neurosurgery, Rostock University Medical Center, Rostock, Germany; Department of Neurosurgery, University Hospital Bonn, Venusberg-Campus 1, Bonn, Germany; Department of Neurosurgery, Rostock University Medical Center, Rostock, Germany; Department of Neurosurgery, Charité-Universitätsmedizin Berlin, Germany; German Cancer Research Center (DKFZ), German Cancer Consortium (DKTK), partner site Berlin, Heidelberg, Germany; Department of Neurosurgery, University Hospital Bonn, Venusberg-Campus 1, Bonn, Germany; Department of Neurosurgery, University Hospital Bonn, Venusberg-Campus 1, Bonn, Germany; Department of Neurosurgery, University Hospital Bonn, Venusberg-Campus 1, Bonn, Germany; Department of Neurosurgery, Rostock University Medical Center, Rostock, Germany; Department of Neurosurgery, Charité-Universitätsmedizin Berlin, Germany; German Cancer Research Center (DKFZ), German Cancer Consortium (DKTK), partner site Berlin, Heidelberg, Germany; Department of Neurosurgery, Charité-Universitätsmedizin Berlin, Germany; Department of Neurosurgery, Rostock University Medical Center, Rostock, Germany; Department of Neurosurgery, Rostock University Medical Center, Rostock, Germany; Department of Neurosurgery, Rostock University Medical Center, Rostock, Germany

**Keywords:** brain metastases, GPA score, infratentorial, multicenter study, prognosis, supratentorial, surgery, surgical resection, survival analysis, survival

## Abstract

**Purpose:**

Infratentorial brain metastases (BMs) pose distinct clinical challenges, but their prognostic relevance remains unclear. This study evaluated the impact of infratentorial location on survival after surgical resection.

**Methods:**

A total of 1,434 patients who underwent surgery for BMs between 2016 and 2023 at three German neurosurgical centers were screened. After applying exclusion criteria, 921 patients were included in the final analysis. Survival outcomes were compared between patients with supratentorial and infratentorial metastases, and prognostic factors for poor outcome were analyzed.

**Results:**

The median age was 63 years (IQR 55–71), and 50.3% were male. The most common primary tumors were NSCLC (51.6%) followed by breast cancer (14.9%) and melanoma (13.5%). Infratentorial metastases were present in 29.5% of patients and were associated with significantly higher rates of hydrocephalus (19.9% vs. 4.2%, OR 5.7, *p *< 0.001) and shorter postoperative survival (7.6 vs. 9.0 months, OR 1.22, *p *= 0.014). Multivariate analysis confirmed infratentorial location as an independent predictor for poor survival (OR 0.65, *p *= 0.015), along with Graded Prognostic Assessment score (OR 1.74, *p *= 0.01), gastric carcinoma histology (OR 0.32, *p *= 0.036), and lack of adjuvant therapy (OR 0.98, *p *= 0.001).

**Conclusion:**

Infratentorial BMs are linked to worse outcome after surgery and should be considered as an independent adverse prognostic factor. Incorporating anatomical location into prognostic models may improve risk stratification and guide clinical decision-making.

Key Points

- Infratentorial location independently predicts reduced postoperative survival.
- Breast cancer metastases more frequently involve infratentorial regions.
- Incorporating tumor location into GPA models may improve survival prediction.

Importance of the Study

Infratentorial brain metastases pose distinct surgical and clinical challenges due to the confined anatomy of the posterior fossa and the risk of hydrocephalus or brainstem compression. While previous studies were limited by small sample sizes, this large multicenter analysis identifies infratentorial location as an independent predictor of poor postoperative survival. These findings emphasize the prognostic relevance of tumor location and support its integration into future GPA-based risk models. Improved understanding of infratentorial disease behavior may guide surgical decision-making and individualized treatment strategies for patients with brain metastases.

Advances in cancer screening, neuroimaging, and multimodal therapies have progressively improved both survival and ­quality of life for patients with systemic malignancies.^1^ Despite these advances, cerebral metastases remain a major cause of neurological dysfunction, disability, and death. It is estimated that 20–40% of patients with solid tumors will develop brain metastases (BMs) during the course of their disease, often requiring adaptation of oncological treatment strategies and limiting eligibility for clinical trials.^2–4^ Among patients with BMs, more than half were aged 65 years or older. Lung cancer was the most common primary tumor, accounting for 61.6% of cases, followed by breast cancer (12%) and malignant melanoma (6.4%).^5^ Among patients with BMs, approximately 30–40% are synchronous and 60–70% metachronous, though proportions vary by tumor type and study methodology.^6^^,^^7^

Up to 25% of BMs involve infratentorial anatomical structures.^8^ Due to their anatomical location, these lesions often produce symptoms distinct from those of supratentorial metastases, manifesting as general neurological signs such as headache, ataxia, and nausea or vomiting.^9^ Infratentorial metastases may also rapidly cause obstructive hydrocephalus and/or brainstem compression, resulting in acute neurological deterioration.^10^ Despite these clinical and neurosurgical challenges, metastases to the infratentorial compartment have not been considered separately and are not included as an adverse prognostic factor in scoring systems such as the Graded Prognostic Assessment (GPA). While the GPA incorporates factors like age, Karnofsky performance status (KPS), number of BMs, and presence of extracranial metastases, it does not account for the anatomical location of brain lesions, such as infratentorial involvement, which may influence patient outcomes.^11^^,^^12^

Taken together, these observations highlight an unmet need to clarify the prognostic significance of infratentorial BMs after surgical resection. This study therefore aimed to determine whether infratentorial location independently predicts worse survival and to quantify its impact on patient outcomes.

## Materials and Methods

### Patient Selection

Between 2016 and 2023, a total of 1434 patients who underwent surgical resection of BMs at the Departments of Neurosurgery of the University Hospital Rostock, University Hospital Bonn, and Charité-Universitätsmedizin Berlin were identified from the institutional electronic databases. The exclusion criteria were: missing magnetic resonance imaging (MRI) scans, lack of histopathological confirmation of the metastasis, no histology-based classification according to the GPA score or loss of follow-up data. Surgical indications comprised neurological deficits, metastasis size unsuitable for radiotherapy. Except for emergency cases, all surgical indications were determined and approved preoperatively by an interdisciplinary tumor board.^13^

### Data Collection

The following data were collected from the institutional electronic databases and outpatient follow-up visits: age, sex, primary tumor type, histopathological diagnosis, KPS, neurological symptoms at presentation, tumor size, localization (infratentorial vs. supratentorial), presence of hydrocephalus, type and date of postoperative complications, as well as oncological treatment modalities including neoadjuvant and adjuvant therapies. Follow-up outcomes were also systematically recorded. Preoperative tumor volume was determined on contrast-enhanced T1-weighted MRI sequences. The largest orthogonal diameters (A, B, and C) were measured manually, and tumor volume was calculated using the standard ellipsoid formula ((A*B*C)/2).

### Aim of the Study

The primary aim of this study was to compare postoperative overall survival (PO-OS) between patients with infratentorial and supratentorial BMs. PO-OS was defined as the time from surgical resection to either the date of death or the date of last follow-up for patients still alive (postoperative overall survival (PO-OS)). In addition, the median PO-OS of the entire cohort was used as a cutoff to distinguish short-term from long-term survivors. Based on this classification, independent predictors of survival were analyzed.

### Statistics

OS was analyzed using Kaplan–Meier curves with log-rank testing and Cox proportional hazards regression. In addition, patients were stratified into short-term and long-term survivors based on median postoperative survival. Comparisons between these groups were performed using univariate and multivariate logistic regression analyses, with results reported as odds ratios (ORs) and 95% CIs. Variables with *p* values < 0.05 in univariate analyses were entered into the respective multivariate models, and statistical significance was defined as *p *< 0.05. Descriptive statistics were applied to summarize patient and treatment characteristics. Statistical analyses were performed using GraphPad Prism 10 (GraphPad Software, California, USA) for descriptive statistics and Kaplan–Meier analyses, and IBM SPSS Statistics version 29 (IBM Corp., Armonk, NY, USA) for multivariate regression analyses.

## Results

**Figure 1.**
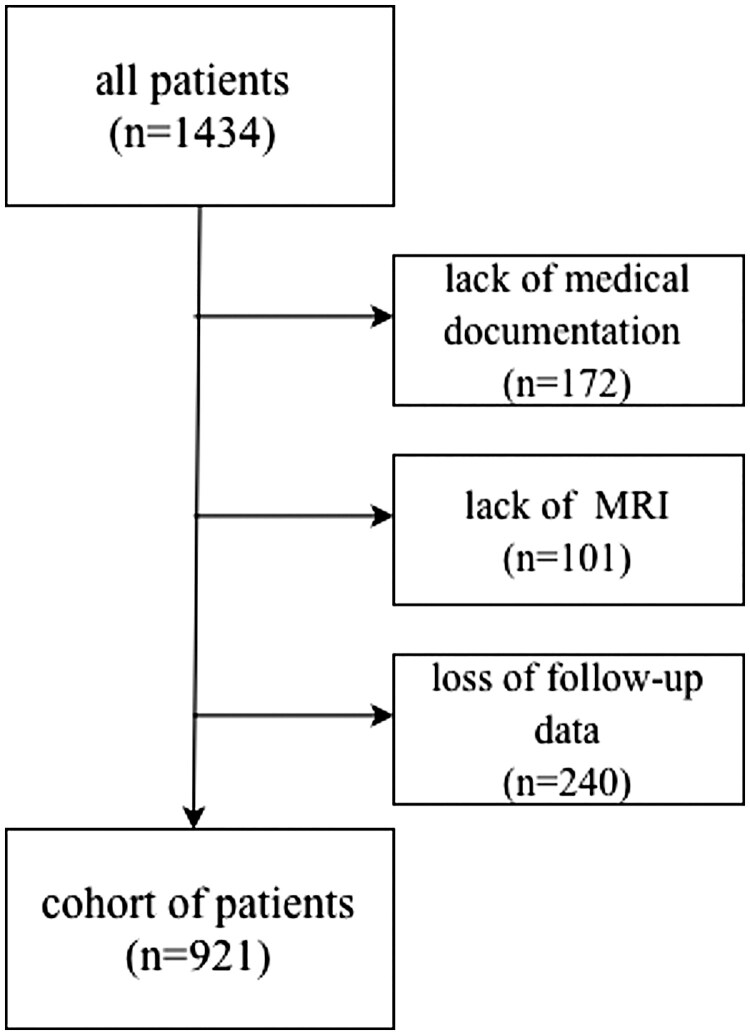
Flowchart of exclusion of patients from research groups.

Out of 1,434 screened patients, 921 patients (64.2%) met the inclusion criteria and were included in the final analysis (Figure 1).  The median age was 63 years (IQR 55–71) and 463 patients (50.3%) were male. A single brain metastasis was present in 431 cases (46.8%). The median tumor volume was 13 cm³ (IQR 5.9–26.2). Hydrocephalus occurred in 81 patients (8.8%). The median postoperative KPS was 80 (IQR 70–90), and the median GPA score was 2 (IQR 2–3). The most common histology was non-small cell lung cancer (NSCLC), diagnosed in 475 patients (51.6%), followed by breast cancer in 137 (14.9%) and melanoma in 124 (13.5%). Postoperative complications occurred in 92 cases (9.9%), with intracranial infections, including sub/epidural empyema and brain abscess, being the most frequent (44 cases, 4.7%). Median postoperative overall survival (PO-OS) after surgery was 8.7 months (IQR 3.8–18.8). The 1-year PO-OS rate was 59.9% in the supratentorial group compared with 53.3% in the infratentorial group, while the 2-year PO-OS rate was 43.9% and 38.9%, respectively (Table 1).

**Table 1.** Baseline clinical characteristics of the study cohort at the time of diagnosis of cerebral metastases, including subgroup division into supratentorial and infratentorial groups.

			Supratentorial group	Infratentorial group	Univariate	
	*Count*	921	649 (70.5)	272 (29.5)	OR 95% CI	*p*—value
Sex,		463 (50.3)	327 (50.4)	136 (50)	1.02 (0.77–1.35)	.486
Male, *n* (%)						
Age, median (IQR)		63 (55–71)	64 (55.6–71.1)	62 (53.5–70.9)	–	.088
GPA, median (IQR)		2 (2–3)	2 (1.5–3)	2 (1.5–3)	–	.296
*n* (%)	<1	40 (4.3)	29 (4.5)	11 (4)	1.11 (0.54–2.26)	.464
	1–2	449 (48.8)	311 (47.9)	138 (50.8)	0.89 (0.67–1.18)	.239
	2.5–3.5	410 (44.5)	294 (45.3)	116 (42.6)	1.11 (0.84–1.48)	.468
	4	22 (2.4)	15 (2.3)	7 (2.6)	0.89 (0.36–2.22)	.486
Primary site of cancer, n (%)	NSCLC	475 (51.6)	334 (51.4)	141 (51.8)	0.98 (0.74–1.31)	.487
	breast cancer	137 (14.9)	86 (13.3)	51 (18.8)	1.51 (1.03–2.21)	**.022**
	melanoma	124 (13.5)	102 (15.7)	22 (8.1)	2.11 (1.31–3.44)	**<.001**
	RCC	60 (6.5)	45 (6.9)	15 (5.5)	1.28 (0.69–2.33)	.261
	SCLC	56 (6)	38 (58.6)	18 (6.6)	0.88 (0.49–1.56)	.379
	Others	69 (7.5)	44 (6.8)	25 (9.1)	0.72 (0.43–1.19)	.129
Postoperative KPS (IQR)		80 (70–90)	80 (70–90)	80 (70–90)	–	.66
*n* (%)	90–100	328 (35.6)	233 (35.9)	95 (34.9)	1.04 (0.78–1.41)	.419
	70–80	475 (51.6)	333 (51.3)	142 (52.2)	0.96 (0.73–1.28)	.431
	<70	118 (12.8)	83 (12.8)	35 (12.9)	0.99 (0.65–1.51)	.525
Systemic treatment before brain metastasis, *n* (%)		390 (42.3)	266 (40.6)	124 (45.6)	0.83 (0.62–1.10)	.112
Initial diagnosis through cerebral metastasis, *n* (%)		342 (37.1)	242 (37.3)	100 (36.7)	1.02 (0.76–1.37)	.471
Neoadjuvant therapy, *n* (%)		115 (12.5)	84 (12.9)	31 (11.4%)	1.15 (0.75–1.79)	.298
-Stereotactic radiosurgery, *n* (%)		19 (16.5%)	15 (17.9%)	4 (12.9)	1.58 (0.52–4.82)	.294
Adjuvant therapy, *n* (%)		540 (58.6)	381 (58.7)	159 (58.5)	1.01 (0.76–1.34)	.501
-Stereotactic radiosurgery, *n* (%)		207 (38.3%)	150 (39.4%)	57 (35.8%)	1.133 (0.80–1.59)	.266
Volumen cm^3^, median (IQR)		11.8 (5.7–23.8)	11.93 (5.5–25.1)	11 (6.0–21.7)	–	.642
Hydrocephalus, *n* (%)		81 (8.8)	27 (4.2)	54 (19.9)	5.7 (3.51–9.28)	**<.001**
VP shunt, *n* (%)		11 (11.9)	0 (0)	11 (40.4)	–	**<.001**
Perioperative complications, *n* (%)		92 (9.9)	58 (8.9)	34 (12.5)	0.68 (0.43–1.07)	.065
	Abscess	44 (4.7)	28 (4.3)	16 (5.8)	0.72 (0.38–1.35)	.196
	Impaired wound healing	27 (2.9)	19 (2.9)	8 (2.9)	0.99 (0.43–2.3)	.569
	Hemorrhage	7 (0.8)	3 (0.5)	4 (1.5)	0.31 (0.07–1.39)	.119
	CSF leak	9 (0.9)	3 (0.5)	6 (2.2)	4.85 (1.21–19.56)	**.022**
	Osteomyelitis	5 (0.5)	5 (0.7)	0	–	.172
PO-SO, median (IQR)		8.7 (3.8–18.8)	9 (4–20.7)	7.6 (3–14.1)	1.22 (1.02–1.47)	**.014**
	1-year PO-OS	57.9%	59.9%	53.3%	–	–
	2-year PO-OS	42.4%	43.9%	38.9%	–	–

Abbreviations: OR: odds ratio; IQR: interquartile range; GPA : Graded Prognostic Assessment; KPS : Karnofsky Performance Status; OS-PO: postoperative overall survival.

### Supratentorial Versus Infratentorial Metastases

Patients were stratified into two groups based on the location of the dominant metastasis requiring surgical resection: supratentorial (*n *= 649, 70.5%) and infratentorial (*n *= 272, 29.5%). Patients with infratentorial disease were slightly younger than those with supratentorial metastases (median age 62 years, IQR 53.5–70.9 vs. 64 years, IQR 55.6–71.1), although the difference was not statistically significant (*p =* 0.88). GPA scores were comparable between the groups (median 2 [IQR 2–3] vs. 2 [IQR 1.5–3], *p =* 0.296). Similarly, the distribution of GPA subgroups was balanced (GPA <1: 4.5% vs. 4.0%; GPA 1–2: 47.9% vs. 50.8%). The postoperative KPS was 80 (IQR 70–90) in both groups. No significant differences were observed regarding adjuvant therapy (58.7% vs. 58.5%) or neoadjuvant therapy (12.9% vs. 11.4%). Median tumor volume was similar between groups (11.9 cm³, IQR 5.5–25.1 vs. 11 cm³, IQR 6–21.7, *p =* 0.642) showing balanced baseline clinical characteristics between supra- and infratentorial group.

Hydrocephalus was significantly more frequent in the infratentorial group (54 cases, 19.9%) compared with the supratentorial group (27 cases, 4.2%) (OR 5.7, *p* < 0.001). Median postoperative OS was significantly shorter in patients with infratentorial metastases (7.6 months, IQR 3–14.1) compared with those with supratentorial lesions (9 months, IQR 4-20.7) (OR 1.22, *p =* 0.014) (Table 1).

**Figure 2.**
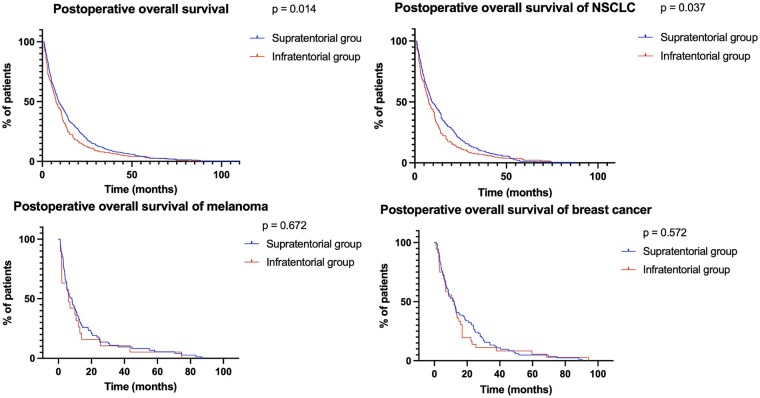
Postoperative overall survival according to lesion location. (a) Entire cohort: infratentorial (*n *= 190) vs. supratentorial (*n *= 455) metastases (*p* = 0.014). (b) NSCLC subgroup infratentorial (*n *= 80) vs. supratentorial (*n *= 259) metastases (*p* = 0.037). (c) Breast cancer subgroup infratentorial (*n *= 37) vs. supratentorial (*n *= 67) metastases (*p* = 0.572). (d) Melanoma subgroup infratentorial (*n *= 36) vs. supratentorial (*n *= 90) metastases (*p* = 0.672).

Kaplan-Meier analysis revealed significantly shorter overall survival in the infratentorial cohort compared with the supratentorial group (*p =* .014). Subgroup analysis showed that this effect was significant in patients with NSCLC (*p =* .037), whereas no significant differences were observed in breast cancer (*p =* .572) or melanoma (*p =* .672) (Figure 2).

### Risk Factors for Poor Outcome

Patients were dichotomized according to the PO-OS of 8.7 months into a poor-prognosis group (*n *= 413; 44.8%) and a favorable-prognosis group (*n *= 508, 55.2%). Patients with poor prognosis were significantly older (median age 66 years, IQR 56.6–73 vs. 63.1 years, IQR 53–68.5; *p *< 0.001) and had lower GPA scores (median 2 [IQR 1.5–2.5] vs. 2.5 [IQR 2–3], *p* < 0.001). Gastric carcinoma was significantly more common in the poor-prognosis group (3.9% vs. 1.8%, *p *= 0.036). Although the median KPS was 80 in both groups, the distribution differed significantly (*p* < 0.001). Adjuvant treatment was less frequently administered in the poor-prognosis group (50.1% vs. 65.6%, *p* < 0.001). Tumor volume was larger in patients with poor outcomes (13 cm³, IQR 5.9–26.2 vs. 10.5 cm³, IQR 5.5–21.2) and multiple BMs were also more frequently observed in this group (*p =* 0.001). Of note, infratentorial tumor location was significantly associated with poor prognosis (33.2% vs. 26.6%, *p =* 0.017) (Table 2).

**Table 2.** Comparison of clinical and treatment characteristics between patients with short-term survival (< 8.7 months) and long-term survival (≥ 8.7 months) following surgery for brain metastases.

		Short-term survivors	Long-term survivors	Univariate		Multivariate	
	Count	413	508	OR 95% CI	*p-*Value	OR 95% CI	*p-*Value
Sex, Male, *n* (%)		220 (53.3)	243 (47.8)	1.24 (0.96–1.61)	.057		
Age, median (IQR)		66 (56.5–73)	63.1 (53–68.5)	–	**<.0001**	0.98 (0.97–0.99)	**<.001**
GPA, median (IQR)		2 (1.5–2.5)	2.5 (2–3)		**<.0001**	1.74 (1.14–2.65)	**.01**
*n* (%)	<1	32 (7.7)	8 (1.6)	5.25 (2.39–11.52)	**<.0001**		
	1–2	239 (57.9)	210 (41.3)	1.94 (1.49–2.53)	**<.0001**		
	2.5–3.5	138 (33.4)	272 (53.5)	0.43 (0.33–0.57)	**<.0001**		
	4	4 (1)	18 (3.6)	0.26 (0.08–0.79)	**.008**		
Primary site of cancer, *n* (%)	NSCLC	206 (49.8)	269 (52.9)	0.88 (0.68–1.15)	.194		
	Breast	58 (14)	79 (15.6)	0.88 (0.61–1.28)	.293		
	Melanoma	54 (13.4)	70 (13.8)	0.94 (0.64–1.37)	.416		
	RCC	30 (7.3)	30 (5.9)	1.24 (0.74–2.1)	.242		
	SCLC	24 (5.7)	32 (6.2)	0.91 (0.53–1.58)	.434		
	CRC	19 (4.5)	16 (3.2)	1.48 (0.75–2.92)	.165		
	Gastric cancer	16 (3.9)	9 (1.8)	2.23 (0.97–5.11)	.041	0.32 (0.11–0.93)	**.036**
	Others	6 (1.4)	3 (0.6)	2.48 (0.61–9.98)	.162		
Postoperative KPS (IQR)		80 (70–80)	80 (70–90)	–	**<.0001**	1.03 (1.01–1.04)	**<.001**
n (%)	90–100	99 (23.9)	229 (45)	2.6 (1.96–3.46)	**<.0001**		
	70–80	240 (58.1)	235 (46.3)	1.6 (1.24–2.09)	**<.0002**		
	<70	74 (18)	44 (8.7)	2.3 (1.54–3.43)	**<.0001**		
Systemic treatment before brain metastasis, *n* (%)		185 (44.8)	205 (40.4)	1.19 (0.92–1.55)	.098		
Initial diagnosis through cerebral metastasis, *n* (%)		141 (34.1)	201 (39.6)	0.79 (0.6–1.03)	.051		
Neoadjuvant therapy, *n* (%)		55 (13.3)	60 (11.8)	1.14 (0.77–1.69)	.277		
-Stereotactic radiosurgery, *n* (%)		10 (18.2)	9 (15)	1.37 (0.55–3.42)	.322		
Adjuvant therapy, *n* (%)		207 (50.1)	333 (65.6)	0.52 (0.4–0.69)	**<.0001**	0.98 (0.97–0.99)	**<.001**
-Stereotactic radiosurgery, *n* (%)		75 (36.2)	132 (39.6)	0.63 (0.45–0.86)	**.002**	0.63 (0.52–0.91)	**.017**
Volumen cm^3^, median (IQR)		13 (5.9–26.2)	10.5 (5.5–21.2)	–	**.031**	0.99 (0.98–1.0)	.42
Hydrocephalus, *n* (%)		44 (10.7)	37 (7.3)	1.51 (0.96–2.39)	**.046**	0.88 (0.59–1.32)	.54
VP shunt, *n* (%)		7 (1.7)	4 (0.8)	2.17 (0.63–7.47)	.169		
Perioperative complications, *n* (%)		32 (7.7)	60 (11.8)	0.63 (0.40–0.98)	**.025**		
Abscess		15 (3.6)	29 (5.7)	0.62 (0.32–1.17)	.09		
Impaired wound healing		8 (1.9)	19 (2.4)	0.5 (0.22–1.17)	.076		
Hemorrhage		4 (0.96)	3 (0.6)	1.64 (0.36–7.39)	.39		
CSF leak		4 (0.96)	5 (1)	0.98 (0.27–3.68)	.62		
Osteomyelitis		1 (0.2)	4 (0.78)	0.3 (0.034–2.74)	.26		
Total number of brain metastases, *n* (%)		1 (0–2)	0 (0–2)		**<.0001**	0.75 (0.62–0.89)	**.001**
one		162 (39.2)	269 (52.9)	0.57 (0.44–0.74)	**<.0001**		
two		104 (25.2)	109 (21.5)	1.23 (0.9–1.67)	.104		
more than two		147 (35.6)	130 (25.6)	1.6 (1.21–2.13)	**.0006**		
Location, *n* (%)							
with a dominant infratentorial metastasis		137 (33.2)	135 (26.6)	1.37 (1.03–1.8)	**.017**	0.65 (0.46–0.92)	**.015**

GPA: Graded Prognostic Assessment; KPS: Karnofsky Performance Status.

Multivariate Cox regression identified several independent predictors of reduced survival: older age (*p* < 0.001, OR 95% CI: 0.98 (0.97–0.99)), lower GPA score (*p =* 0.01, OR 95% CI: 1.74 (1.14–2.65)) gastric carcinoma histology (*p* = 0.036, OR 95% CI: 0.32 (0.11–0.93)), lower KPS (*p* < 0.001, OR 95% CI: 1.03 (1.01–1.04)), absence of adjuvant treatment (*p* < 0.001, OR 95% CI: 0.98 (0.97–0.99)), multiple BMs (*p =* 0.001, OR 95% CI: 0.75 (0.62–0.89)), and infratentorial tumor location (*p =* 0.015, OR 95% CI: 0.65 (0.46–0.92)).

## Discussion

This study investigated the prognostic relevance of infratentorial BMs in a large multicenter surgical cohort. When comparing patients with dominant infratentorial and supratentorial metastases, no significant differences were observed in tumor volume, GPA score, age, or KPS, indicating that the two groups were generally well balanced. Notably, breast cancer patients more frequently presented with dominant infratentorial lesions requiring surgery, whereas melanoma patients more often had supratentorial involvement. The key finding was that infratentorial location was significantly associated with a higher incidence of preoperative hydrocephalus and reduced postoperative overall survival. Multivariate analysis confirmed that infratentorial location remained an independent adverse prognostic factor even after adjustment for age, GPA score, KPS, tumor histology, multiplicity of metastases, and adjuvant treatment. Infratentorial tumors were also more likely to require cerebrospinal fluid diversion procedures and carried a higher risk of postoperative CSF leakage, although the overall complication rate did not differ between groups. Importantly, survival was significantly worse in the infratentorial group, with a median OS of 7.6 months compared with 9 months in supratentorial patients, as well as reduced 1- and 2-year survival rates. Differences of this magnitude may still be clinically meaningful in patients with advanced metastatic disease. Such differences may influence patient counseling, perioperative risk assessment, and multidisciplinary treatment planning.

In the study by Steinruecke et al., 85 patients were analyzed, and although infratentorial metastases were associated with a lower median survival, the difference did not reach statistical significance (median OS 323 days vs. 277 days; *p *= 0.06). However, patients with infratentorial lesions had more frequently experienced postoperative surgical complications, which occurred in up to 25% of cases.^14^ Similarly, Hamed and colleagues investigated 245 patients and, using matched-pair analysis (61 vs. 61 patients), found a nonsignificant difference in postoperative survival (13 vs. 11 months).^8^ On the other hand, Enders et al. demonstrated in a cohort of 114 NSCLC patients that beyond established GPA parameters such as KPS and extracranial metastases, infratentorial location was independently associated with worse survival.^15^ Similarly, Chaichana and colleagues reported on 708 patients, including 140 (19.8%) who underwent surgical treatment for cerebellar metastases, and found a significantly reduced survival in the infratentorial group (median OS 8.2 vs. 9.9 months; *p *= 0.04 in multivariate analysis).^16^ In our study, the overall survival was significantly shorter in the infratentorial cohort compared with the supratentorial group as well. In subgroup analyses, this effect was pronounced in patients with NSCLC, which represented the largest subgroup in our cohort. By contrast, although breast cancer and melanoma patients with infratentorial metastases demonstrated a trend toward reduced survival, these differences did not reach statistical significance (*p* = 0.572 and *p *= 0.672, respectively). We interpret this discrepancy as a consequence of limited sample size in the non-NSCLC subgroups, which likely reduced statistical power despite a visible survival difference. The lack of information on extent of resection represents a relevant limitation, especially for infratentorial lesions where subtotal resection may be more common due to anatomical constraints.

When patients were stratified by postoperative survival, we confirmed several key prognostic factors. In line with prior studies, GPA proved to be a robust predictor, as did its individual components: age, number of BMs, and KPS. In terms of histology, gastric carcinoma was again associated with significantly worse outcomes. Previous reports have similarly emphasized that gastrointestinal metastases, while rare, are linked to particularly poor survival.^17^^,^^18^ Nevertheless, in many cases, surgical resection remains the only feasible treatment option for space-occupying infratentorial metastases. Prior work has shown that surgery can improve median survival compared with radiotherapy alone, underscoring its therapeutic relevance in this challenging subgroup.^19^ Most importantly, infratentorial location of the dominant metastasis emerged as a statistically significant risk factor for shorter postoperative survival in both univariate and multivariate analysis. Thus, although the GPA remains a strong prognostic tool, our data indicate that tumor location provides additional prognostic information and may refine risk stratification within a surgically treated population.

Kraus and colleagues analyzed 235 patients with infratentorial metastases and observed postoperative hydrocephalus in 18.5%—a rate comparable to our 19.9%. Importantly, they identified hydrocephalus as an independent predictor for poor prognosis.^10^ In our study, while hydrocephalus was significant in the univariate analysis, it did not remain significance in multivariate analysis, likely due to collinearity with infratentorial location, whereby one variable may have displaced the other. In this context, ventriculoperitoneal shunting represents an important treatment option for patients with persistent hydrocephalus. However, the potential risk of peritoneal tumor dissemination remains a matter of debate. While isolated case reports have described peritoneal carcinomatosis following shunt placement, larger series have failed to confirm this association, suggesting that the risk is likely overestimated.^20^

Further, patients with prolonged survival experienced more postoperative complications. We attribute this paradox to the fact that many complications occur in a delayed postoperative period, which shorter-surviving patients may not live long enough to develop.

Taken together, infratentorial metastases represent a distinct clinical challenge due to the limited space of the posterior fossa, risk of brainstem compression, and frequent obstructive hydrocephalus requiring urgent neurosurgical intervention. Earlier studies often failed to demonstrate statistically significant survival differences, likely owing to limited sample sizes; in contrast, our large surgical cohort shows that infratentorial location is not only associated with specific complications but also independently predicts reduced postoperative survival.

Our findings further indicate that infratentorial location provides additional prognostic information within a surgically treated population. However, it remains premature to propose a formal modification of established GPA-based models, which were developed for unselected patients with brain metastases and would require validation in non-surgical cohorts.^11^^,^^12^ Nevertheless, our data support a hypothesis-generating concept that infratentorial involvement—particularly when accompanied by symptomatic mass effect or obstructive hydrocephalus—may represent an adverse prognostic dimension not fully captured by current GPA parameters. Conceptually, this could correspond to a modest reduction in prognostic weighting (e.g., 0.5–1.0 points), depending on the presence and severity of hydrocephalus. Further prospective studies integrating surgical and non-surgical populations are warranted.

## Conclusion

Infratentorial BMs are linked to worse outcomes after surgery and should be considered as an independent adverse prognostic factor. Incorporating anatomical location into prognostic models may improve risk stratification and guide clinical decision-making. These findings highlight the need for tailored surgical strategies and integration of multimodal treatment in this high-risk subgroup.

## Limitations

This study has several limitations. First, its retrospective design inherently introduces the risk of selection and information bias and limits the ability to establish causal relationships between tumor location and survival. Second, the multicenter nature of the cohort implies heterogeneity in surgical techniques, perioperative management, and adjuvant treatment strategies, which may have influenced outcomes despite standardized data collection. Third, although we included a large number of patients, subgroup analyses—especially for less frequent primary tumors such as breast cancer and melanoma—were likely underpowered to detect small but clinically meaningful survival differences. Fourth, the interval between surgery and postoperative radiotherapy as well as the specific radiotherapy modalities were not consistently documented, potentially confounding survival analyses. Fifth, the extent of resection could not be evaluated, as this parameter was not systematically recorded across all centers; this may affect outcome interpretation, particularly for lesions in eloquent infratentorial regions where subtotal resection is more common. Sixth, the treatment strategies for BMs are highly heterogeneous and often multimodal, including surgery, stereotactic radiosurgery, whole-brain radiotherapy, and systemic therapies. The present analysis focuses exclusively on surgically treated patients, and the indication for surgery inherently introduces a selection bias that may limit generalizability to non-surgical populations. Finally, despite careful efforts to maintain data completeness, missing follow-up information for some patients could have introduced bias in survival estimates.

## Data Availability

The data presented in this study are available on request from the corresponding author.
